# Palliative Treatment of Atresia of the Vagina and Uterus

**Published:** 1881-12

**Authors:** 


### PALLIATIVE TREATMENT OF ATRESIA OF THE VAGINA AND UTERUS.

In the course of a clinical lecture on Atresia, Dr. A. F. Erich of Baltimore (*Atlanta Med. Register*, Nov., 1881), speaks of palliative treatment as follows:

Palliative measures in atresia have heretofore been limited to the relief of pain at the menstrual period, and no one, so far as I am aware, has proposed anything short of an operation for the complete relief of the terrible suffering. The success which I have had, however, in a case where an operation was absolutely refused, prompts me to urge the trial, in suitable cases, of the bromide of potassium. The case I refer to is one of congenital atresia of the vagina. The patient is a young lady of 20 years of age. Menstrual molimina first showed themselves at the age of 15. The abdomen began to enlarge, and she suffered intensely at every menstrual period, requiring large doses of morphia to control the pain. She has been in my charge for three years, and now requires no morphia or other anodynes. About a week before the beginning of each menstrual period she begins taking half-drachm doses of potassium bromide three times a day. This has the effect of much reducing the hyperæmia of the reproductive organs and so limiting the amount of blood poured out. In the interval between two periods sufficient of the fluid portion of the blood is absorbed to keep the tumor from growing larger. In fact, if any change is noticeable, it is that the tumor is growing smaller rather than increasing. Furthermore, she suffers very little, if any more discomfort at her menstrual periods than thousands of other women who regard themselves in perfect health. A decided objection to an operation, especially in an unmarried subject, is the great difficulty of keeping the newly formed vagina pervious. The canal having no mucous lining the opposed surfaces have a strong tendency to re-unite, which necessitates the constant wearing of the vaginal plug. This becomes, at length, very irksome and leads in the end to neglect of this precaution, resulting almost certainly in the closure of the canal. It might be worth while to try the palliative treatment in cases where an operation seems either unnecessary, exceptionally difficult, or fraught with danger to life.

